# Factors Influencing Antimicrobial Choice and Duration During the Last Month of Life in Hospitalized Patients

**DOI:** 10.1093/ofid/ofaf670

**Published:** 2025-11-03

**Authors:** Fergal Howley, Eva Jones, Ciara Anderson, Ryan Fagan, Sam Grennan, Bettina Korn, Liam Townsend, Ciaran Bannan, Aoibheann Conneely

**Affiliations:** Department of Infectious Diseases, St James's Hospital, Dublin, Ireland; Department of Palliative Medicine, St James's Hospital, Dublin, Ireland; Department of Infectious Diseases, St James's Hospital, Dublin, Ireland; Department of General Medicine, St James's Hospital, Dublin, Ireland; Department of Infectious Diseases, St James's Hospital, Dublin, Ireland; Department of Nursing, St James's Hospital, Dublin, Ireland; Department of Infectious Diseases, St James's Hospital, Dublin, Ireland; Department of Clinical Medicine, Trinity College Dublin, Dublin, Ireland; Department of Infectious Diseases, St James's Hospital, Dublin, Ireland; Department of Clinical Medicine, Trinity College Dublin, Dublin, Ireland; Department of Palliative Medicine, St James's Hospital, Dublin, Ireland; Department of Clinical Medicine, Trinity College Dublin, Dublin, Ireland; Our Lady's Hospice and Care Service, Dublin, Ireland

**Keywords:** antimicrobial stewardship, end-of-life care, palliative care

## Abstract

**Background:**

Antimicrobial prescribing in patients approaching end of life is complex. We aimed to describe antimicrobial use in the final 4 weeks of life among hospitalized patients and identify factors associated with antimicrobial consumption.

**Methods:**

We conducted a retrospective review of antimicrobial use among inpatients during their last 4 weeks of life in a tertiary Irish hospital. Data collected included antimicrobial agents and duration of therapy, microbiological sampling, infection services input, palliative care services input, patient demographics, frailty scores, and markers of inflammation. Univariate analysis and multivariable linear regression were used to assess factors associated with antimicrobial-free time before death.

**Results:**

Two hundred and fifty-eight patients were included, with 91% receiving antimicrobials during the study period. Only 36% had a presumed infection at the time of admission. Antimicrobial use was characterized by broad-spectrum agents, did not correlate with culture results, and had a median duration (interquartile range) of 10.5 (5–18) days. Palliative care consultation (*P* = .004) and longer length of stay (*P* < .0001) were associated with a longer antimicrobial-free interval before death. Within the antimicrobial cohort, 26% developed acute kidney injury. Cessation of antimicrobial therapy often occurred late, with 40% of patients receiving antimicrobial therapy within 24 hours of death.

**Conclusions:**

There was a high burden of antimicrobial use in patients nearing the end of life, characterized by broad-spectrum, empiric therapy, often continued until hours before death. We recommend expansion of antimicrobial surveillance and collaboration between infection and palliative care services in order to optimize and rationalize antimicrobial prescribing in patients approaching end of life.

## Teaser

Antimicrobials were prescribed in the four weeks preceding death in 91% of included patients. Antimicrobial usage was not guided by microbiology results nor by input from infection specialists. Palliative care input was associated with increased antimicrobial-free time prior to death.

Antimicrobial prescribing in patients approaching end of life is complex. Perceived benefits of symptom control and prolonging life must be balanced against risk of adverse drug reactions, intravenous access, therapeutic drug monitoring, and patient discomfort, as well as the wider issues of antimicrobial resistance and stewardship. Factors including patient and family preference, cultural or religious beliefs, and physician antimicrobial prescribing practices at end of life can all influence antimicrobial use in this patient cohort [1]. Physician decision remains the strongest influence on initiating antimicrobial therapy in the palliative context, while cessation or withdrawal of same is more complex, involving multiple parties [2].

Evidence for the use of antimicrobials in patients approaching end of life varies, with reported benefit in certain infections, but no benefit, or even harm, in other conditions, while variability in methodology and patient cohorts poses a challenge in drawing general conclusions [3, 4]. Consistent with reported interstudy variation, antimicrobial use at the end of life varies widely across regions, settings, and specialties, ranging from 4% to 97.5% [5].

There are no evidence-based guidelines for the use of antibiotics in patients approaching end of life, though state-of-the-art reviews and good-practice guidelines have been published [6].

Though 44% of deaths in Ireland occur in acute hospitals, data regarding antimicrobial use in patients approaching end of life in an Irish acute hospital setting are lacking [7]. We aimed to examine antimicrobial usage in the last 4 weeks of life for patients who were deemed not suitable for end-organ support, either through invasive ventilation, cardiopulmonary resuscitation, or administration of vasopressors, at the largest tertiary hospital in Ireland. Factors influencing antimicrobial prescribing in this cohort were examined, including patient characteristics, treating specialty, laboratory and microbiological data, and input from infection and palliative care services. Additionally, development of antimicrobial-associated toxicities was also recorded. Identification of factors influencing prescribing habits at the end of life will inform the development of guidelines and modulate clinical practice.

## METHODS

### Study Setting and Participants

This study was conducted at St James's Hospital, Dublin, Ireland, a tertiary-level health care center. A retrospective review of all patients who died during January to June 2023 was conducted. This period was chosen to mitigate for seasonal bias in prescribing habits. A treatment escalation plan guides the level of life-sustaining medical interventions that occurs for a patient. It should be a shared care plan between treating clinicians and the patient and family. In our institution, treatment escalation plans have 3 escalation categories: (1) full escalation of care including cardiopulmonary resuscitation, (2) active ward-based care with selected interventions and not for cardiopulmonary resuscitation, and (3) comfort-focused care. All treatment escalation plans in our institution have a field for documentation of an antimicrobial plan; however, this is not mandatory. We included all patients who died with either an “active ward-based” or “comfort-focused care” treatment escalation plan in place at the time of death. We performed further analysis on those who had received antimicrobial therapy within the last 28 days of life.

Exclusion criteria included (1) patients who died in the intensive care unit (ICU), (2) patients who died in the emergency department, (3) patients who died within 24 hours of admission to the hospital, (4) patients who died who were for full escalation of care at the time of death, and (5) patients <18 years of age. Deaths in the ICU were excluded as these are cases where medical care is escalated and very active until the point of withdrawal of care, after which death frequently occurs very rapidly [8, 9]. These are considered a different cohort of deaths compared with those deaths that occur on the wards where there have been some discussions about limitations of medical interventions. Patients who had spent a period of time in the ICU before being transferred to a ward prior to death were included, assuming they did not meet the exclusion criteria above. Patients who were for full escalation of care at the time of death including intubation and ventilation, or cardiopulmonary resuscitation, were considered “unexpected” deaths and hence excluded.

### Clinical Covariates

Demographic data (age, sex) and medical background (comorbidities, comedications, Rockwood Clinical Frailty Scale score) together with admission-related factors (reason for admission and admitting specialty) were recorded. Admitting specialty was categorized as medical, surgical, and hematological/oncological. Data on antimicrobial use (agent, duration, and route of administration), cause of death, infection specialist and palliative care input, and resuscitation status were also extracted from electronic medical records. Changes to antimicrobial therapy were recorded, and broadening of antimicrobial coverage was defined as switching to an antimicrobial with an extended spectrum of activity or addition of another antimicrobial agent to increase bacterial or fungal coverage. Laboratory results including sputum, urine, and blood cultures, as well as C-reactive protein (CRP) and white blood cell (WBC) count results at admission and at peak, were also included. Nasopharyngeal polymerase chain reaction (PCR) samples for severe acute respiratory syndrome coronavirus 2 (SARS-CoV-2), respiratory syncytial virus (RSV), and influenza A/B were recorded, as were intercurrent stool sample analysis and associated *Clostridioides difficile* rates. Formed stool samples do not undergo analysis at our institution. The sending of a stool sample is a surrogate marker of loose stool. Development of acute kidney injury during the final 4 weeks, as defined by a rise in serum creatinine of ≥50% of baseline, was also recorded. Screening swabs for multidrug-resistant organisms were recorded. These include nasal, axilla, and groin swabs for methicillin-resistant *Staphylococcus aureus* (MRSA) and rectal swabs for extended-spectrum β-lactamase–producing *Enterobacterales* (ESBL), carbapenemase-producing *Enterobacteriaceae* (CPE), and vancomycin-resistant *Enterococci* (VRE). Antimicrobial use in the community or on prior admissions occurring within the last 4 weeks of life was not captured.

Specialist infection service input was recorded only if the patient was reviewed by the infectious diseases (ID) or antimicrobial stewardship (AMS) team, as these services routinely document advice on electronic patient records. Input from these teams is not mandated in our institution. ID input is usually through consult request from the primary team and involves a face-to-face review of the patient. AMS input does not require formal consultation and is typically provided for patients receiving broad-spectrum antimicrobial therapy (eg, carbapenems) and other patients highlighted as warranting review by a dedicated AMS pharmacist. AMS input involves a virtual review and documentation of recommendations through the electronic patient records. It is not mandatory for AMS recommendations to be followed by the treating team.

### Statistical Analysis

Descriptive statistics are reported as means with standard deviations and medians with interquartile ranges (IQRs), as appropriate. Univariate analysis using Spearman's rank correlation coefficient and subsequent Cox regression analysis were used to assess factors associated with antimicrobial-free time before death. Bonferroni correction for multiple testing was applied to the univariate analysis. Significant variables on univariate analysis as well as factors identified a priori as being associated with antimicrobial prescribing, namely input from an infection service and MDRO carriage, were included in Cox regression models. Statistical significance was set at the .05 level. All analyses were performed using Stata, version 18.0 (Stata Statistical Software).

## RESULTS

### Cohort Characteristics

**Figure 1.**
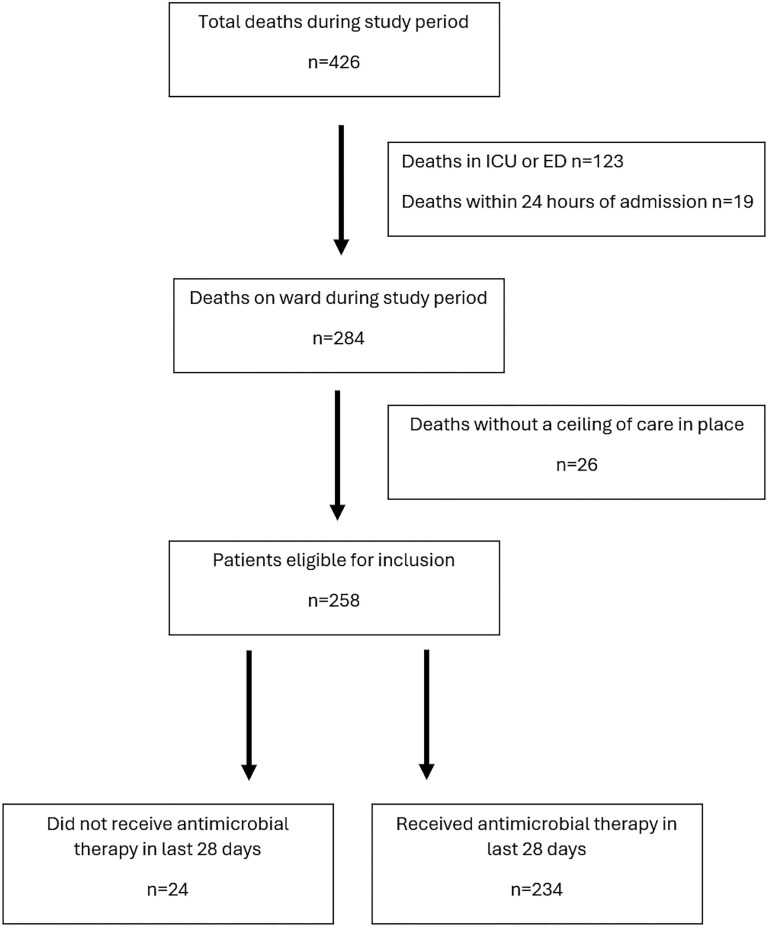
Patient enrollment diagram illustrating deaths during the study period, patients meeting exclusion criteria, and eligible patients who received antimicrobial therapy. Abbreviations: ED, emergency department; ICU, intensive care unit.

Four hundred and twenty-six patients died in St James's Hospital between January and June 2023. Of these, n = 123 were excluded as they died in the intensive care unit or emergency department. A further n = 19 were excluded due to having died within 24 hours of admission. Among the remaining n = 284 patients, n = 26 were for full escalation of care at the time of death and were excluded, resulting in n = 258 patients included for analysis. There were n = 20 patients transferred from other hospitals included in the overall cohort. Of the n = 258 who met eligibility criteria, n = 24 (9%) did not receive any antimicrobials in the 28 days preceding death. Full enrollment details are shown in Figure 1. The median age of the cohort (IQR) was 82 (16) years, while the median length of stay (IQR) was 18 (31) days. The majority were admitted under medical specialities. Complete cohort characteristics are shown in Supplementary Table 1.

### Patterns of Antimicrobial Usage in the Last Four Weeks of Life

**Figure 2.**
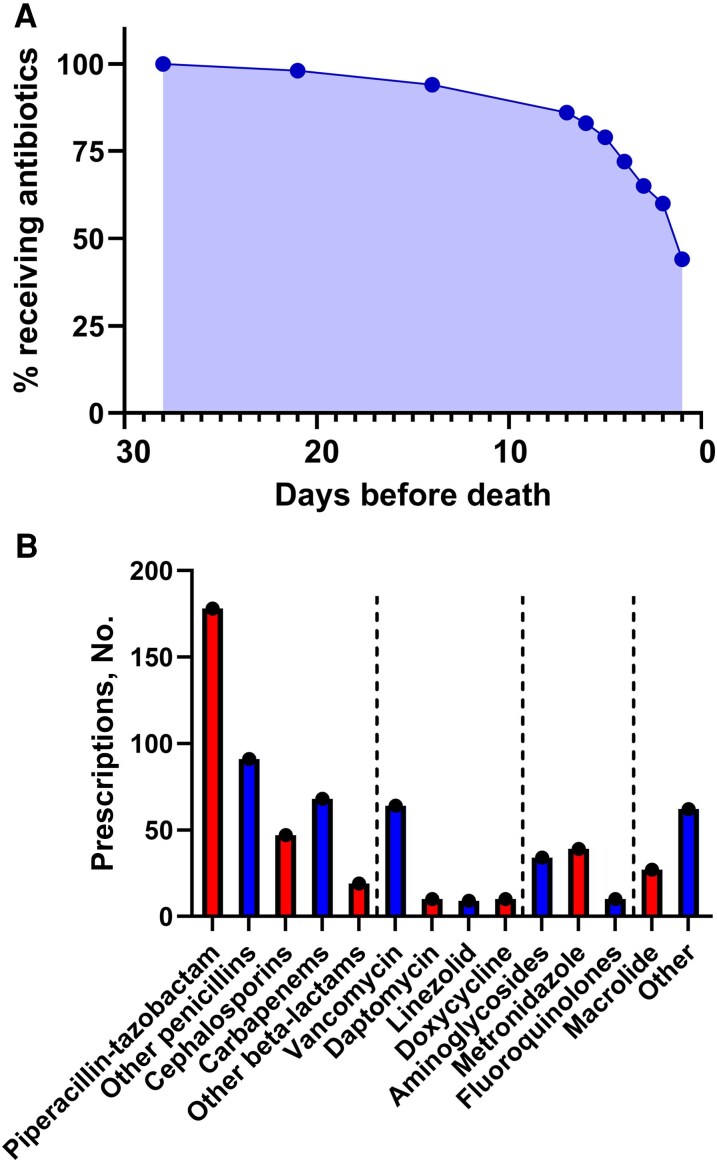
Timing and choice of antimicrobial agent. *A*, Percentage of patients receiving antimicrobials in the days preceding death. *B*, Antimicrobial agents prescribed in the final 4 weeks of life. Dashed lines group antimicrobials into broad classes: penicillins, cephalosporins, and carbapenems; gram-positive agents; gram-negative and anaerobic agents; atypical and other agents.

Of the n = 234 patients who received antimicrobial therapy in the final 4 weeks of life, the median duration of treatment (IQR) was 10.5 (5–18) days. Forty percent of patients (n = 94) were deemed to have an infectious diagnosis of some description by the admitting physician and were initiated on antimicrobial therapy upon admission. This included n = 8 transferred from external hospitals who were already established on antimicrobial therapy. The most common infection source was respiratory (72/94), with genito-urinary source in n = 9, skin and soft tissue in n = 5, intra-abdominal in n = 3, and nonspecified in n = 5. The median time from last dose of antibiotics to death was 39.5 hours. Of the n = 258 total cohort, 104 patients (40%) received antibiotics within 24 hours of death, while the majority (n = 201, 78%) received antibiotics in their final 7 days of life (Figure 2*A*).

There was a wide range of antimicrobials used during the study period, with 668 instances of antimicrobial agents being prescribed. Piperacillin-tazobactam was the most commonly prescribed antimicrobial, followed by other penicillin agents. Carbapenems were prescribed in 68/234 (29%) patients who received antibiotics. The antimicrobials used are shown in Figure 2*B*. A change in antimicrobial therapy occurred in 142 patients (61%) during the last 4 weeks of life. Most changes (131/142, 92%) represented escalation of therapy, defined as either the addition of an antibacterial or antifungal agent or a switch of antimicrobial therapy from an agent with a narrower spectrum of activity to an agent with a broader spectrum of activity. Almost all patients (231/234, 99%) received intravenous antimicrobial therapy during this time period. Indication for antimicrobial therapy was documented in 194 (83%) cases, as outlined in Supplementary Table 2. The treatment escalation plan included reference to antimicrobial therapy in only 24 (10%) cases. Among n = 185 who were documented as being for “comfort-focused care” at the time of death, 37 (20%) received antimicrobial therapy after documentation of futility.

### Diagnostic Sampling, Specialist Input, and Potential Toxicity

**Figure 3.**
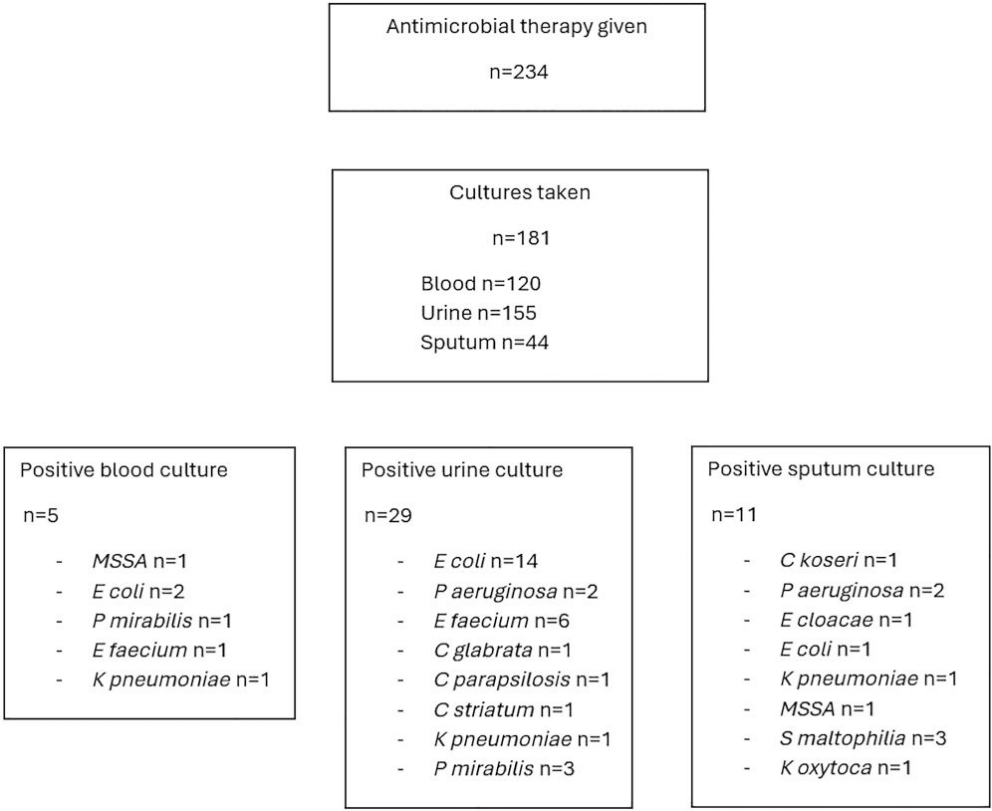
Microbiology culture outcomes demonstrating the number of patients receiving antimicrobial therapy, the number of patients who underwent microbiological sampling, and positive culture results. Abbreviation: MSSA, methicillin-sensitive *Staphylococcus aureus*.

Multidrug-resistant organism carriage was identified in n = 49 (19%) individuals: n = 10 MRSA, n = 25 VRE, n = 6 ESBL-producing gram-negative bacteria, and n = 8 other (including CPE). Culture samples from blood, urine, and/or sputum were taken in n = 181 (77%) of those who received antibiotics, with n = 120 having blood cultures taken. Blood cultures were taken in n = 71 (71/120, 59%) before the administration of antibiotics, with a further n = 37 (37/120, 31%) having blood cultures drawn within the first 24 hours after antibiotic administration. Blood cultures were obtained >24 hours after the first antimicrobial dose in n = 12 (12/120, 10%); 3 of these were transfers from external institutions already established on antimicrobial therapy. Excluding presumptive contaminants (such as coagulase-negative *Staphylococcus* species in blood or yeast in urine or sputum), a positive culture result was found in 5/120 (4.1%) blood cultures, 29/155 (19%) urine cultures, and 11/44 (25%) sputum cultures (Figure 3). Of the 45 clinically significant cultures, 8 (18%) were MDROs (4 ESBL, 3 VRE, 1 CPE). Nasopharyngeal swabs for viral PCR were taken in 188 patients, with SARS-CoV-2 detected in n = 29 and RSV detected in n = 3.

Antimicrobial therapy was reviewed by specialist infection services in 33/234 cases (14%) and was considered inappropriate in 19/33 (58%) cases, due to incorrect duration (n = 13), no indication for therapy (n = 6), and inappropriate agent (n = 7).

Stool samples were sent in n = 96 patients receiving antibiotics (96/234, 41%). Six samples were PCR and toxin positive for *C. difficile,* resulting in a 3% incidence of *C. difficile* in the patient population receiving antibiotics. Acute kidney injury was observed in n = 61 (61/258, 24%) individuals from the total cohort. There was a significantly higher rate of acute kidney injury in the cohort receiving antimicrobials (60/234, 26%) compared with the cohort that did not receive antimicrobials (1/24, 4%; χ^2^ = 5.56; *P* = .02).

### Factors Influencing Time From Last Antimicrobial Dose to Death

In the n = 234 patients who received antimicrobial therapy, the median time from last dose to death (IQR) was 39.5 (109) hours. The factors associated with time between antibiotic cessation and death were investigated. On univariate analysis, palliative care input and increased length of stay were both associated with prolonged antimicrobial-free time before death. Input from infection services, laboratory values associated with infection (CRP and WBC count), and microbiological results were not associated with antimicrobial-free time (Table 1).

**Table 1.** Factors Associated With Discontinuation of Antimicrobials

	Spearman's Rho (ρ)	*P* Value
Patient factors		
Age	0.04	.56
Sex	0.09	.89
Comorbidity count	0.03	.70
Clinical frailty score	−0.03	.68
Admission factors		
Palliative care consult	0.33	<.0001
Infection specialist input	0.01	.89
Admitting team	−0.06	.39
Length of stay	0.41	<.0001
Laboratory factors		
WBC at admission	−0.07	.41
WBC peak	−0.06	.34
CRP at admission	−0.08	.32
CRP peak	−0.11	.10
Any positive culture	−0.07	.37
MDRO colonization	−0.11	.08

Bonferroni correction for multiple testing applied.

Abbreviations: CRP, C-reactive protein; MDRO, multidrug-resistant organism; WBC, total white blood cell count.

These factors were investigated further with a multivariable Cox regression model including significant variables on univariate analysis as well as relevant variables chosen a priori (input from infection specialists and colonization with an MDRO). Following adjustment, both palliative care input and length of total hospital stay remained significantly associated with increased antimicrobial-free time before death on multivariable analysis (Table 2).

**Table 2.** Predictors of Antimicrobial-Free Time Before Death

	Hazard Ratio (95% CI)	*P* Value
Palliative care consult	0.51 (0.38–0.68)	<.0001
Length of stay	0.99 (0.98–0.99)	<.0001
Infection service input	0.86 (0.59–1.26)	.45
MDRO colonization	1.21 (0.87–1.68)	.25

Multivariable Cox regression, with all variables included in the model shown.

Abbreviation: MDRO, multidrug-resistant organism.

## DISCUSSION

We demonstrate high rates of antimicrobial use in patients approaching end of life in a tertiary Irish hospital, disproportionate to the number of patients with a working diagnosis of active infection. Additionally, antimicrobial usage was not driven by microbiological results. Respiratory tract infections were the most common indication for treatment, similar to prior research in this area [10]. Piperacillin-tazobactam was the most commonly prescribed antibiotic. Use of broad-spectrum antibiotics and escalation of therapy, including the use of carbapenems, were common.

Of the 33 cases (14%) where specialist ID input was documented, the antimicrobial was deemed inappropriate in a majority of cases. This mirrors data suggesting that antimicrobial use is often inappropriate in the end-of-life setting [11]. The low rate of specialist input highlights the need for proactive antimicrobial stewardship in this cohort, rather than the current consult-based model. Previous studies on antimicrobial usage at end of life have demonstrated a tendency toward initiation, and continuation, of antibiotics even in cases of apparent futility, highlighting the need for improved training of nonspecialists in palliative care skills [12]. This may provide insight into why antimicrobial consumption in this patient cohort remains so high, despite AMS approaches already in existence, such as pre-authorization strategies [13]. Simple stewardship interventions include incorporating antimicrobial use into discussions about goals of care, early involvement of palliative care services if death is anticipated, and education of physicians and pharmacists on how to rationalize antimicrobial use; these interventions may improve antimicrobial prescribing in this cohort [14]. In addition to this, we propose an increased role for ID specialists in reviewing patients approaching end of life and active involvement in discussions around goals of care. Divergence of antimicrobial prescribing from culture results in the absence of proactive antimicrobial stewardship was also seen during the coronavirus disease 2019 (COVID-19) pandemic, with antibiotics often continued despite absence of evidence to support bacterial infection [15, 16]. Interestingly, surrogates of infection such as CRP and WBC count did not influence antimicrobial prescribing in our cohort. The utility of these markers is a topic of debate. A scale assessing factors influencing antimicrobial prescribing in end-of-life care demonstrated that physicians give symptoms a higher weight than factors related to infection, including markers of inflammation, when deciding whether to initiate antibiotics [17]. This may explain our findings that surrogate markers of infection did not influence antimicrobial prescribing.

Comparison with international data is challenging due to heterogeneity of study designs, but the rate of antibiotic use reported here was higher than the 59.6% described in a Korean study, in which third-generation cephalosporins were the most commonly prescribed antibiotics [18]. Antimicrobial prescribing among patients for comfort-focused care has been reported at 15.6% in America and 33.7% in Australia [3, 19].

Antimicrobial therapy at end of life is not without complications. Patients with advanced illness who are in receipt of treatment, and older adults in particular, may experience significant drug toxicity and adverse drug events related to antimicrobials [20, 21]. This is evidenced by the 3% incidence of *C. difficile* in our cohort receiving antimicrobial therapy. Additionally, a stool sample was sent in 41% of patients in receipt of antimicrobials, suggestive of a large burden of diarrheal illness in this cohort. While these gastrointestinal symptoms are multifactorial, antimicrobials are well-recognized contributors [22]. There was also a significantly higher incidence of kidney injury in the cohort receiving antimicrobials. Kidney injury is well recognized as a contributor to symptom burden at end of life [23]. Despite these toxicities, physician attitudes often favor continuing antimicrobial therapy, with 41% of physicians reporting “sometimes” or “often” continuing therapy when patients are moving to comfort-focused care, with patient or family preference, along with symptom control, cited as common reasons for continuation [24]. Prolonged empiric antimicrobial therapy has broader consequences for the development of antimicrobial resistance, with longer antimicrobial treatment courses associated with increased carriage of resistant organisms [25, 26].

We found that longer length of stay was associated with a longer antimicrobial-free period before death. This reflects the findings of a Peruvian study of 239 deaths in an acute hospital, in which 81.5% of patients received antimicrobial therapy until death, and length of stay was associated with earlier discontinuation before death [27]. We also found palliative care service input to be associated with reduced antimicrobial use in the days before death. We hypothesize that this may be explained by the primary team recognizing signs of dying in their patients and responding by stopping therapy and consulting palliative care services, though this was not captured in our data. This identifies a clear need for increased education among treating physicians on recognizing failure of therapy, treatment futility, and recognizing the dying phase. Uncertainty around end-of-life prognosis and challenges around shared decision-making have previously been cited as factors contributing to ongoing antimicrobial usage in the palliative setting [28]. This was reflected in our finding that just 10% of treatment escalation plan documentation made specific reference to antimicrobial therapy, despite evidence that documentation of patient preference for limited antimicrobials reduces antimicrobial use toward the end of life [29]. Though our hospital's treatment escalation plan does include a field specifically for antimicrobial prescribing, this was rarely completed. We propose that physicians discuss antimicrobial prescribing when establishing goals of care, in a similar manner to discussions around the role of ICU transfer, cardiopulmonary resuscitation, and other invasive interventions.

There are several limitations to this study. First, it was conducted in an acute hospital with well-established palliative care, ID, and antimicrobial stewardship services, so it may not reflect practices across other Irish centers. Additionally, it is retrospective in nature, and causal relationships between factors analyzed and antimicrobial prescribing cannot be established. It did not evaluate the attitudes of patients, caregivers, or prescribers toward antimicrobials at the end of life.

This study demonstrates a high rate of broad-spectrum antimicrobial prescribing at the end of life, despite limited objective evidence to support infection. It identifies areas for intervention to improve antibiotic use in this area, which may improve patient comfort, reduce adverse events, and slow the development of antimicrobial resistance.

## CONCLUSIONS

Broad-spectrum, empiric antimicrobial consumption in the final weeks of life is high at our center, reflecting international trends. Considering the risk of adverse drug reactions and toxicity and limited evidence supporting their use in end-of-life care, this represents an opportunity to optimize patient comfort toward the end of life, while also reducing antimicrobial consumption, with potential benefits in antimicrobial stewardship as well as health care and environmental costs. There is a need for further research to define the role for antimicrobial therapy in patients approaching the end of life in order to guide decision-making and antimicrobial stewardship approaches. We suggest a need for increased education of physicians in discussing and documenting patient preferences regarding antimicrobial use at end of life and in the recognition of dying. We suggest a need for increased AMS input among patients approaching end of life and collaboration between palliative care and infection specialists to optimize pathways for antimicrobial rationalization in patients approaching end of life.

## Supplementary Material

ofaf670_Supplementary_Data

## Supplementary Data

Supplementary materials are available at *Open Forum Infectious Diseases* online. Consisting of data provided by the authors to benefit the reader, the posted materials are not copyedited and are the sole responsibility of the authors, so questions or comments should be addressed to the corresponding author.
