# Supplementary material for: Factors Influencing Antimicrobial Choice and Duration During the Last Month of Life in Hospitalized Patients
Source: Open Forum Infect Dis. 2025 Nov 3;12(11):ofaf670. doi: 10.1093/ofid/ofaf670 (PMC12626221; doi:10.1093/ofid/ofaf670)
Supplement: ofaf670_Supplementary_Data [file ofaf670_supplementary_data.zip › Supplemental table 2.docx]

**Supplemental Table 2: Indications for antimicrobial prescribing**

| Indication | Number of episodes (%) | Indication | Number of episodes (%) |
| --- | --- | --- | --- |
| Respiratory | 125 (64) | Abdominal | 19 (10) |
| SSTI/bone/joint | 11 (6) | Genitourinary | 15 (8) |
| Other | 7 (4) | Uncertain source | 17 (9) |
