# Supplementary material for: Factors Influencing Antimicrobial Choice and Duration During the Last Month of Life in Hospitalized Patients
Source: Open Forum Infect Dis. 2025 Nov 3;12(11):ofaf670. doi: 10.1093/ofid/ofaf670 (PMC12626221; doi:10.1093/ofid/ofaf670)
Supplement: ofaf670_Supplementary_Data [file ofaf670_supplementary_data.zip › Supplemental_Table1_updated.docx]

**Supplemental Table 1: Cohort characteristics**

|  | Total cohort  (n=258) |
| --- | --- |
| Age, years; median (IQR) | 82 (16) |
| Sex, female; n (%) | 135 (52) |
| Nursing home resident; n (%) | 15 (6) |
| Clinical frailty score; mean (SD) | 5 (2) |
| Co-morbidity count, n; median (IQR) | 5 (4) |
| Co-medication count, n; median (IQR) | 8 (6) |
| WBC at admission, x10^9^; median (IQR) | 10.8 (7) |
| Peak WBC, x10^9^; median (IQR) | 17 (10) |
| CRP at admission, mg/L; median (IQR) | 64 (133) |
| Peak CRP, mg/L; median (IQR) | 154 (138) |
| Specialty; n (%)  -medical  -surgical  -haemonc | 194 (75)  21 (8)  43 (17) |
| Length of stay, days; median (IQR) | 18 (31) |
| Reason for admission, n (%)   - Respiratory - Cardiac - Frailty - Gastrointestinal - Hepatology - Neurological - Oncological - Renal - Surgical - Other | 67 (26)  24 (9)  32 (12)  15 (18)  12 (5)  30 (12)  29 (23)  13 (5)  21 (8)  15 (6) |
| Infection at admission, yes; n (%) | 94 (36) |
| Any culture sent; n (%)   - Blood - Urine - Sputum | 185 (72)  123 (48)  158 (61)  45 (17) |
| Any culture positive; n (% of taken)   - Blood - Urine - Sputum | 65 (35)*  7 (6)*  44 (28)*  19 (42)* |
| Clinically significant positive culture (excluding presumptive contaminants); n (% of taken)   - Blood - Urine - Sputum | 45 (25)*  5 (4)*  29 (18)*  11 (24)* |
| Antibiotics prescribed, yes; n (%) | 234 (91) |

*Percentages here differ to those included in the text, as this table includes all 258 patients who met eligibility criteria for inclusion in the study, including those who did not receive antibiotics in the final four weeks of life. By contrast, the percentages included in the text refer only to cultures sent among the patients who received antibiotics in the final four weeks of life.
